# On the Electrochemical Detection of Alpha-Fetoprotein Using Aptamers: DNA Isothermal Amplification Strategies to Improve the Performance of Weak Aptamers

**DOI:** 10.3390/bios10050046

**Published:** 2020-04-30

**Authors:** Ramón Lorenzo-Gómez, Daniel González-Robles, Rebeca Miranda-Castro, Noemí de-los-Santos-Álvarez, María Jesús Lobo-Castañón

**Affiliations:** 1 Departamento de Química Física y Analítica, Universidad de Oviedo, Av. Julián Clavería 8, 33006 Oviedo, Spain; lorenzoramon@uniovi.es (R.L.-G.); dgonrob88@gmail.com (D.G.-R.); mirandarebeca@uniovi.es (R.M.-C.); santosnoemi@uniovi.es (N.d.-l.-S.-Á.); 2 Instituto de Investigación Sanitaria del Principado de Asturias, Avenida de Roma, 33011 Oviedo, Spain

**Keywords:** aptamer, alpha-fetoprotein, dissociation constant, rolling circle amplification, terminal deoxynucleotidyl transferase

## Abstract

Affinity characterization is essential to develop reliable aptamers for tumor biomarker detection. For alpha-fetoprotein (AFP), a biomarker of hepatocellular carcinoma (HCC), two DNA aptamers were described with very different affinity. In this work, we estimate the dissociation constant of both of them by means of a direct assay on magnetic beads modified with AFP and electrochemical detection on carbon screen-printed electrodes (SPCE). Unlike previous works, both aptamers showed similar dissociation constant (K_d_) values, in the subµM range. In order to improve the performance of these aptamers, we proposed the isothermal amplification of the aptamers by both terminal deoxynucleotidyl transferase (TdT) and rolling circle amplification (RCA). Both DNA amplifications improved the sensitivity and also the apparent binding constants from 713 nM to 189 nM for the short aptamer and from 526 nM to 32 nM for the long aptamer. This improvement depends on the true affinity of the binding pair, which ultimately limits the analytical usefulness.

## 1. Introduction

Cancer management is a multifactorial decision-making task that relies on a combination of clinical evidences, biochemical parameters and tumor biomarker values. Ideally, a tumor biomarker is any biological characteristic that is detected only in the presence of cancer. However, the perfect biomarker does not exist, and most approved for clinical usage present variable degrees of false negative (limited sensitivity) or false positive (limited specificity) results. In fact, the cut-off values and recommended use are under continuous scrutiny and could be modified over the years.

This is the case of alpha-fetoprotein (AFP), one of the two biomarkers suitable for large-scale screening in addition to the controversial prostate specific antigen [1]. AFP serum levels rapidly decrease after birth and remain below 10 ng mL^−1^ under healthy conditions except in pregnant women [2,3]. Since the expression of AFP is highly specific for the liver, it was found elevated in hepatocellular carcinoma (HCC) patients and proposed as a tumor biomarker. However, AFP alone is not enough for HCC diagnosis, screening or surveillance [4]. Its suboptimal performance, that is, increased levels in benign liver pathologies and teratomas or low levels in small HCC [5], has prompted that US and European guidelines allow HCC surveillance with or without AFP control, in contrast to Asian guidelines that recommend its use in combination with imaging techniques or even other biomarkers [2]. Nonetheless, it remains as a supplemental test when only low-quality or inconclusive images are available. AFP has also found applicability as a prognostic (disease outcome in the absence of therapy), monitoring of therapy and recurrence biomarker and to identify the best candidates for liver transplantation. The optimal cut-off level for each purpose has not been definitively adopted [4,6]. This means that AFP remains widely employed in the clinical setting in spite of its limitations.

As with other protein biomarkers, in large centralized facilities, the methods of choice for AFP detection are immunoassays performed in automated instruments [7]. To reduce the ever-increasing health expenditures and decentralize the analysis, the extreme success of glucometer for diabetes patients, an electrochemical biosensor, is a model to imitate. To this end, stable, cost-effective and reproducible reagents are needed. Antibodies do not fully meet all these criteria. In contrast, aptamers considered as the chemical antibodies of non-protein nature have demonstrated their better chemical and thermal stability, inherent reproducibility among batches due to chemical synthesis and lower manufacturing costs than antibodies.

The last decade has witnessed the selection of three aptamers for AFP; an RNA aptamer with anti-proliferative activity on HCC cells via the downregulation of AFP expression [8] and two DNA aptamers for diagnostic applications [9,10]. The LOD achieved ranged between 6.97 ag mL^−1^ [11] and 259 ng mL^−1^ [12], for the 72 nt-long aptamer selected by microfluidics [9], and 0.23 pg mL^−1^ [13] to 500 ng mL^−1^ [14], for the 75 nt-long aptamer selected by capillary electrophoresis (CE) [10]. This means that the LODs span over 10 and 6 orders of magnitude, respectively. The lowest values exceed by several orders of magnitude the LOD expected for binding pairs having K_d_ in the nM or subµM range in the absence of any target amplification scheme [15,16]. Nonetheless, some of these reports use other strategies recently reviewed by Gooding et al. to reach ultralow limits of detection [16], mainly through the use of nanostructures to obtain surfaces with high density of receptors. This way, the equilibrium is shifted toward the complex aptamer-target due to the increased chance of rebinding. Additional strategies comprise the restriction of the volume to the nanoscale, the implementation of amplification schemes or the use of magnetic beads to improve the target mass transport. To the best of our knowledge, DNA amplification schemes have not been employed to improve the performance of anti-AFP aptamers.

DNA isothermal amplification methods can be easily implemented in aptamer-based assays because of the nucleic acid nature of the receptor [17,18]. Their aim is to push down the LOD of DNA-based sensors (both aptasensors and genosensors) and to facilitate the full integration of all analytical steps on a single biosensor platform, without requiring additional instrumentation as in PCR-based amplification strategies [19,20,21]. Several isothermal methods have been developed in the last two decades [22]. We have selected two of them: terminal deoxynucleotidyl transferase (TdT) and rolling circle amplification (RCA). TdT is a non-templated elongation of DNA by its 3′ end that allows the incorporation of labeled nucleotides in a tunable ratio [23,24]. RCA benefits from the quickness and high fidelity of phi29 polymerase to copy thousands of times the sequence of a pre-ligated circular DNA (known as padlock), thus providing multiple binding sites for reporting probes [25]. While TdT DNA elongation of an aptamer is directly performed, RCA requires tagging the aptamer with an RCA primer, a highly specific site for circularizing the padlock and thus triggering the elongation.

In this work, we have studied and compared the binding affinity of the two DNA aptamers evolved against AFP. We used magnetic beads to anchor the protein and aptamers with several tags for conventional enzymatic amplification. Our results support the outperformance of the 72 nt-long aptamer. Then, TdT and RCA were tested in combination with these aptamers. TdT was more efficient in shifting the apparent binding constant toward lower values, while RCA showed superior amplification power.

## 2. Materials and Methods

All reagents and instrumentations are listed in the Supplementary Information. The following protocols were used in this work.

### 2.1. Modification of Tosylactivated Magnetic Particles with AFP

Dynabeads^®^ M-280 Tosylactivated were modified according to manufacturer recommendations. Briefly, 5 mg of magnetic beads (MBs) were washed twice with 0.1 M Na-phosphate buffer pH 7.4. Then, 250 µL of 0.1 M Na-phosphate buffer containing 200 µg mL^−1^ human AFP and 1.2 M ammonium sulfate was added to the MBs and incubated for 12–18 h at 37 °C with continuous stirring at 1300 rpm. After removing the supernatant, the unreacted tosyl groups were blocked with a 0.5% BSA solution in 1× PBS incubated at 37 °C for 1 h, under stirring. Then, two washing steps with 1× PBS—0.1% BSA were performed and finally the MBs were resuspended in this BSA-containing buffer to a storage concentration of 20 mg mL^−1^. The modified beads were kept at 4 °C while not in use.

The supernatant from the MBs modification was analyzed by the Bradford assay, applying the protocol recommended by the manufacturer of the Bradford reagent. The amount of immobilized protein was calculated as the subtraction of the amount of AFP found in the supernatant from the amount of AFP used for immobilization.

### 2.2. Binding Assays on AFP-Modified Magnetic Particles

First, 60 µg of AFP-modified MBs were incubated with 250 µL of increasing concentrations of anti-AFP aptamers prepared in 1× PBS, for 30 min at 25 °C, with continuous stirring at 1300 rpm. Then, the supernatant was discarded, and the MBs were washed twice with 1× PBS—0.01% Tween 20 (washing buffer).

**Scheme 1:**
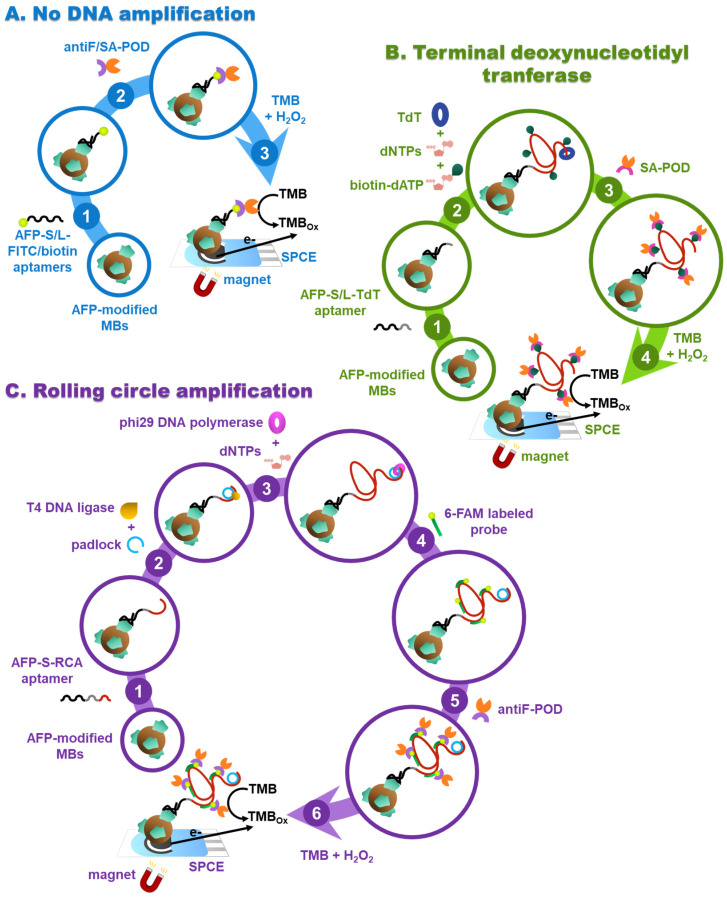
Depiction of the methodology employed in the binding curves on alpha-fetoprotein (AFP)-modified magnetic beads (MBs). (**A**) Without DNA amplification: 1. Aptamer binding; 2. Enzyme labeling with streptavidin-peroxidase (POD) or anti-fluorescein Fab fragment-POD conjugates; 3. MBs magnetic entrapment on screen-printed carbon electrodes (SPCEs), substrate addition, enzymatic reaction and chronoamperometric measurement. (**B**) With terminal deoxynucleotidyl transferase (TdT) amplification: 1. Aptamer binding; 2. TdT elongation; 3. Enzyme labeling with the streptavidin-POD conjugate; 4. MBs magnetic entrapment on SPCEs, substrate addition, enzymatic reaction and chronoamperometric measurement. (**C**) With rolling circle amplification (RCA): 1. Aptamer binding; 2. Padlock annealing and ligation; 3. RCA elongation; 4. Reporter probe hybridization; 5. Enzyme labeling with antiF-POD conjugate; 6. MBs magnetic entrapment on SPCEs, substrate addition, enzymatic reaction and chronoamperometric measurement.

When no nucleic acid amplification was used for signal enhancement, the step following the incubation with anti-AFP aptamers (Table S1) (AFP-S-biotin, AFP-S-FITC or AFP-L-FITC; Scheme 1A, step 1) was the binding of an enzymatic conjugate. A volume of 250 µL of anti-fluorescein-Fab-fragment-peroxidase conjugate (antiF-POD, 0.5 U mL^−1^ in 1× PBS—0.5% casein) or streptavidin-peroxidase (SA-POD, 2.5 µg mL^−1^ in 1× PBS—0.01% Tween 20) was added, depending on the label of the aptamer, and incubated for 30 min at 25 °C, with continuous shaking (Scheme 1A, step 2). After two washing steps with washing buffer and one with 1× PBS, the MBs were resuspended in 30 µL of this latter buffer. For the electrochemical measurement, 10 μL of MBs were dropped on the working electrode of the SPCE with a 4 mm diameter and circular Neodymium magnet placed exactly under it. In this way, MBs are distributed onto the surface and entrapped magnetically for 1 min (Scheme 1A, step 3). Finally, 40 μL of a ready-to-use TMB solution was added, and the enzymatic reaction proceeded for 60 s. Chronoamperometry was carried out immediately after at 0 V for 60 s.

When TdT amplification was used, the incubation with anti-AFP aptamers (AFP-L-TdT or AFP-S-TdT; Scheme 1B, step 1) was followed by the amplification reaction. After two washings with washing buffer, the MBs were resuspended in 50 µL of TdT mix, containing 10 U TdT, 0.25 mM CoCl_2_, 500 µM dNTPs, and 25 µM biotin-dATP prepared in 1× reaction buffer. The reaction proceeded for 1 h at 37 °C, under continuous stirring (Scheme 1B, step 2). Next, the supernatant was discarded, and the MBs were washed twice. The enzymatic labeling with SA-POD (Scheme 1B, step 3), enzymatic reaction of TMB and chronoamperometric measurement (Scheme 1B, step 4) were performed as stated above.

In the case of RCA, the incubation with anti-AFP aptamers (AFP-S-RCA; Scheme 1C, step 1) was followed by the annealing and ligation of the circularizable RCA template (padlock). To this aim, the washed MBs were resuspended in 30 µL of ligation mix, containing 3.75 Weiss T4 DNA ligase and 10 nM padlock in 1× ligase buffer, and incubated for 30 min at 25 °C, under continuous stirring (Scheme 1C, step 2). After two washings, the MBs were then resuspended in 30 µL of RCA mix, containing 7.5 U phi29 DNA polymerase and 500 µM dNTPs in 1× polymerase buffer, and incubated for 15 min at 37 °C, under continuous stirring (Scheme 1C, step 3). Next, the MBs were washed twice and incubated with 250 μL of 100 nM 6-FAM-labeled reporter probe in 1× PBS for 30 min at 25 °C (Scheme 1C, step 4). The enzymatic labeling with antiF-POD (Scheme 1C, step 5), enzymatic reaction of TMB and chronoamperometric measurement (Scheme 1C, step 6) were performed as mentioned above.

### 2.3. TdT Amplification Study by Gel Electrophoresis

TdT amplification was performed in solution, incubating 20 µL of the reaction mix (1.5 µM aptamer, 30 U TdT, 500 µM dATP, and 0–50 µM biotin-dATP prepared in 1× reaction buffer, for 1 h at 37 °C under gentle stirring. After that, 5 µL of 10 µM T40 probe was added, and the mixture was heated at 70 °C for 10 min to deactivate the enzyme. Next, the solution was cooled at room temperature to allow the hybridization between the poliA-tailed aptamer and the T40 probe, resulting in a double stranded DNA. A volume of 5 µL of this product was mixed with 1 µL of 6× loading buffer and loaded in a 2% agarose gel (prepared in 1× TBE containing SimplySafe DNA staining dye). The electrophoresis was run for 45 min under a potential difference of 80 V. DNA bands were revealed under an UV lamp, and the size of the elongated aptamer was estimated by comparison of its displacement on the gel to that of a DNA ladder of 20 bp.

## 3. Results

### 3.1. Comparison of Binding Affinity in the Absence of DNA Amplification

Two DNA aptamers selected against AFP were used in this study: a 72-nt aptamer and the truncated version of a 75-nt aptamer that has been previously tested to keep its affinity without the flanking primer sites [26,27]. For brevity, in this work, we will denote those aptamers as “long” (AFP-L) and “short” (AFP-S), respectively. First, the short aptamer tagged with a biotin (AFP-S-biotin, Table S1) or with a fluorescein (AFP-S-FITC, Table S1) was tested on AFP-modified magnetic beads using increasing concentrations of the aptamer (0.25–2 µM). Direct binding of the aptamer on the beads is revealed through the labeling with a peroxidase conjugate (SA-POD or antiF-POD) and further addition of the TMB substrate. After 1 min, the current of the reduction of the TMB oxidized enzymatically is measured by chronoamperometry.

**Figure 1:**
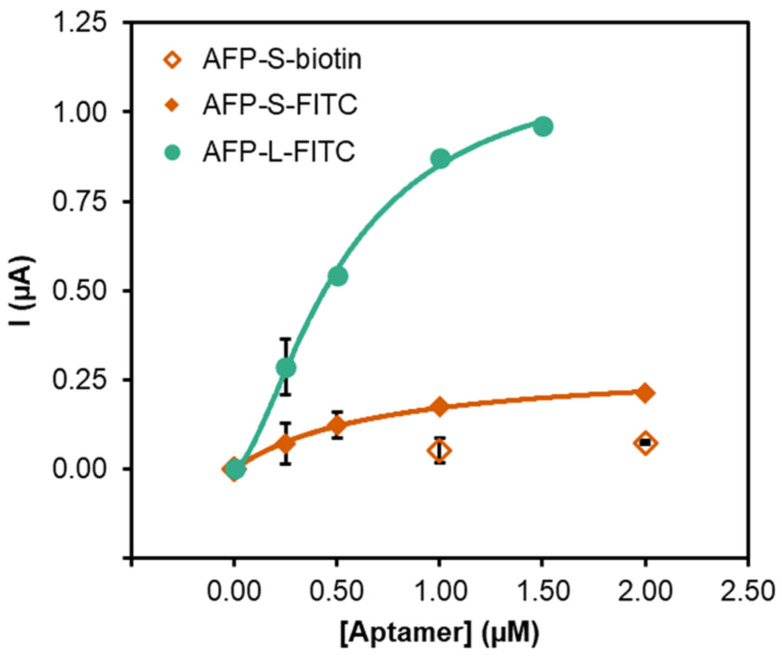
Binding curves obtained with AFP-modified MBs and the biotinylated or fluorescein-tagged short aptamer and the fluorescein-tagged long aptamer.

Figure 1 shows the corresponding binding curves. As expected, the multivalence of streptavidin-POD conjugate reduces the current measured by chronoamperometry in comparison to the monovalent anti-fluorescein Fab fragment-POD, so the former was discarded as the reporter conjugate. Comparatively, the fluorescein-tagged long aptamer showed much higher currents, which indicates that the enzyme activity immobilized on the magnetic beads is higher, and more aptamers bind AFP at each specific concentration. Fitting the binding curves to the Hill equation yields dissociation constant (K_d_) values of 713 ± 63 nM and 526 ± 101 nM, for the short and long aptamers, respectively.

Interestingly, the long aptamer shows certain cooperativity (*n* = 1.6 ± 0.4) while the short one fits very well to a 1:1 stoichiometry (Langmuir model). The K_d_ for the short aptamer is close to the reported for the full sequence. The long aptamer, however, seems to be a much poorer binder than expected. The K_d_ is more than two orders of magnitude higher. It is important to bear in mind that this equilibrium model assumes that target binding does not change the concentration of the ligand in solution (the aptamer here). This may not be true when there is a high packing density of the probes (the protein here) or when working with low sample volumes. In such cases, the binding signal, which is a measurement of the probe occupancy, is no longer related to the ligand concentration in solution but to the relative amount of ligand in solution and probes on the surface. Under those conditions, the midpoint of the binding curve represents half the effective probe concentration [15]. To check whether this regime is operating in the present case, the effective AFP concentration was estimated to be about 34 nM. This value is 20-fold higher than the reported K_d_. This means that depletion of the ligand due to rapid binding might occur, theoretically resulting in an apparent K_d_ of 17 nM (34/2). However, the midpoint of our experimental binding curve is well above this value, which excludes the operation under the ligand-depletion regimen. The reported K_D_ was measured by SPR in a reverse set up where the aptamer was anchored at an unknown density, and the protein was in solution. Accordingly, the discrepancy could arise from limited access of the aptamer to the protein binding site. However, the aptamer was selected on AFP-modified epoxy-MBs, which binds to primary amines and sulfhydryl groups as the tosylactivated MBs herein used, ruling out this explanation. Together, everything points to a “true” K_d_ higher than the reported one.

### 3.2. Amplification by Terminal Deoxynucleotidyl Transferase Elongation

**Figure 2:**
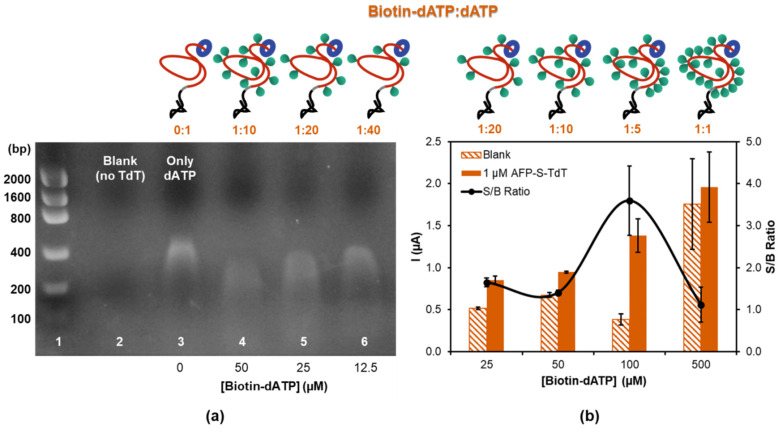
(**a**) Agarose gel electrophoresis of TdT elongation of short aptamer. Lane 1: DNA ladder. Lane 2: TdT blank. TdT elongated product obtained with Lane 3: 100% dATP. Lane 4: 10% biotin-dATP. Lane 5: 5% biotin-dATP. Lane 6: 2.5% biotin-dATP. (**b**) Current obtained in the electrochemical assay with different biotin-dATP:dATP ratios in the absence (blank, striped bars) and in the presence of the short aptamer at 1 µM (solid bars) and the corresponding signal-to-blank (S/B) ratio for each condition.

Isothermal amplification of the aptamer is an effective way of enhancing the sensitivity and shifting the apparent K_d_ to lower ligand concentrations. TdT elongates any ss-DNA provided that a free 3′-OH end is available. In addition to the four natural nucleotides, it incorporates a wide variety of unnatural analogues including ribonucleotides, biotinylated or fluorescent-labeled nucleotides [28]. The rate of incorporation is nucleotide-, ion- and label-dependent [29]. We selected the biotinylated dATP (biotin-dATP) as the labeled nucleotide for subsequent enzyme conjugation using SA-POD. First, we studied the appropriate ratio of biotin-dATP to dATP in order to obtain the longest tail with the optimum number of biotin labels. To this aim, the TdT was first performed in solution and the products visualized on agarose gel. Figure 2a shows the results obtained with the AFP-S-TdT. Note that although a certain length distribution appeared in the elongated products as a consequence of the enzyme random kinetics, their mean size depends on the ratio of biotin-dATP. The higher the biotin-dATP:dATP ratio, the shorter the products are, confirming the preference of the enzyme for the natural nucleotide. About 600 dATP nucleotides can be incorporated in 60 min (Figure 2a, lane 3) while only half this value when 10% of the nucleotides are biotinylated (Figure 2a, lane 4).

In the electrochemical assay, TdT was carried out on AFP-modified MBs after the interaction with 1 µM of the short aptamer. Then the SA-POD conjugate was added, and the reduction of the enzymatic product was measured by chronoamperometry. The number of biotinylated dATP determines the amount of enzymes carried by each aptamer-AFP complex and thus the amplification of the analytical signal. The current increased when the biotin-dATP:dATP ratio was varied between 1:20 and 1:1 (5–50% of biotin-dATP). This increase is related with the higher concentration of labeled nucleotides that can be incorporated to the rising strand (Figure 2b) when the proportion of biotin-dATP increases. The net current multiplies by 3 for a 4-fold increase in biotin-dATP concentration (from 25 to 100 µM), while it dramatically decreases when the concentration further increases 5-fold (from 100 to 500 µM) due to a strong increase in the blank current (no aptamer present). Unspecific binding to the MBs of biotin-dATP might account for this increase of the blank signal. A “trade off” between increasing the reaction rate (by increasing the amount of unlabeled substrates) and the amount of labeled nucleotides needed for sensing with proper reproducibility provides the optimum biotin-dATP:dATP ratio when 5% (1:20) of the nucleotides are biotinylated This ratio was used in subsequent experiments.

**Figure 3:**
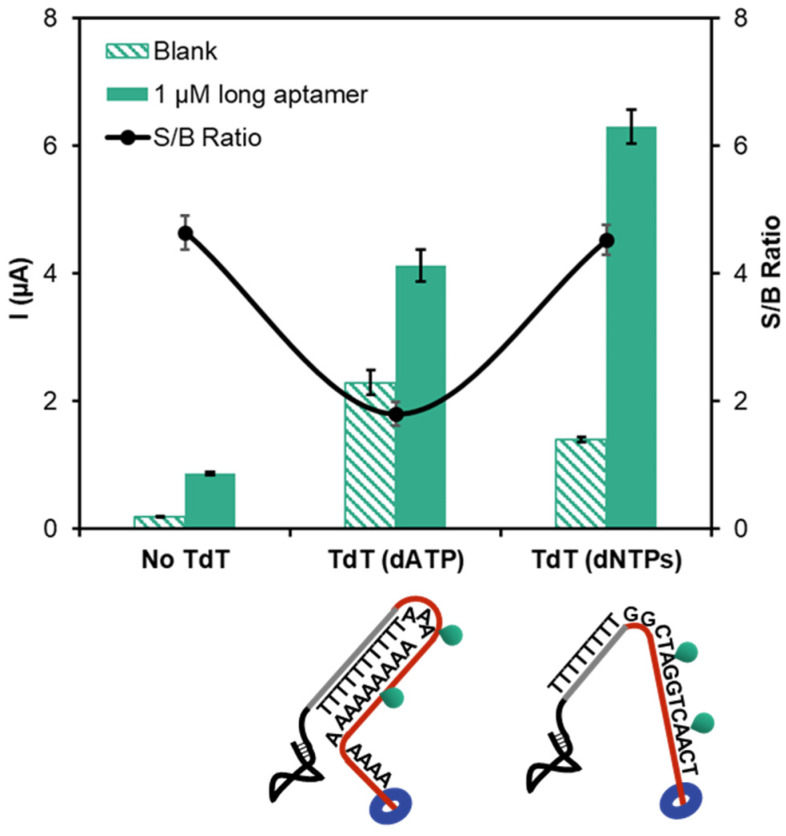
Chronoamperometric currents obtained without and with TdT amplification using only dATP or a mixture of dNTPs at 500 µM concentration in the absence (striped bars) and in the presence (solid bars) of the long aptamer at 1 µM and the corresponding signal-to-blank ratio at each condition.

Since the elongation rate depends on the nucleotide used, we examine the influence of adding just dATP or an equimolar mixture of the four nucleotides (dNTP), maintaining the total concentration. Figure 3 shows that the current dramatically increases when the mixture of dNTPs is employed, suggesting that the TdT is somehow impeded when only dATP is used as a source of unlabeled nucleotides. In this experiment the long aptamer with an 8-thymine tail at the 3′ end was used in order to avoid steric hindrance between the binding event and the enzyme elongation. It has been demonstrated that the formation of duplexes or G-quartets can halt the TdT elongation [30]. In our case, the thymine spacer and the adenine-based nucleotides can hybridize slowing down the reaction as it is schematized in Figure 3. Of note, in the absence of TdT, the current is much lower due to the presence of a single enzyme-labeling site. The amplification power of TdT, in terms of current, by incorporation of multiple enzymes per recognition event is 3.3 and 9-fold for the dATP and dNTP elongations, respectively.

**Figure 4:**
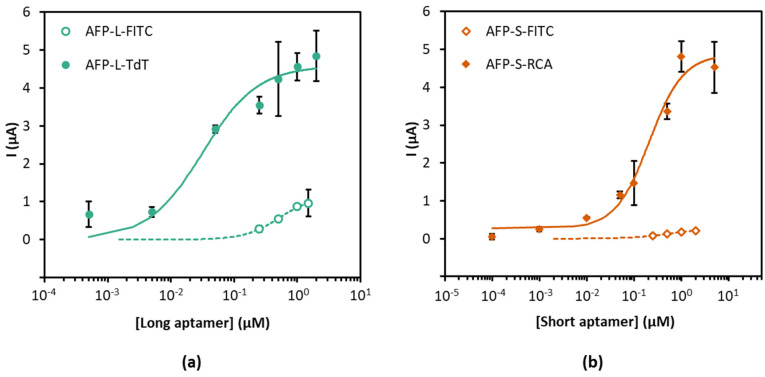
(**a**) Binding curves obtained on AFP-modified magnetic beads with the long aptamer without (open circles) and with (solid circles) TdT amplification. (**b**) Binding curves obtained on AFP-modified magnetic beads with the short aptamer without (open diamonds) and with RCA amplification (solid diamonds).

The effect of TdT elongation on the apparent binding affinity was studied by obtaining the corresponding binding curve on AFP-modified MBs under the optimized conditions with electrochemical detection. Figure 4a compares the TdT-amplified and the non-TdT amplified binding curves obtained with the long aptamer. The dramatic increase in the magnitude of the analytical signal and the displacement of the TdT amplified curve toward smaller concentrations of aptamer are apparent. This is reflected in the apparent K_d_ value estimated from the fitting to the Langmuir equation, 32 ± 11 nM, more than an order of magnitude lower than the above calculation for the non-TdT amplified assay. This value is still an order of magnitude higher than the reported by SPR.

### 3.3. Rolling Circle Amplification

Previously we were able to observe an increase in the apparent affinity of a candidate tumor marker, NGAL, of three orders of magnitude using RCA as isothermal amplification technique [31]. In order to understand whether this improvement is general for any binding pair or it depends on the true affinity, we combined RCA with the short aptamer. RCA requires the design of an appropriate primer that is added to the 3′ end of the aptamer. This primer acts as a complementary strand for the hybridization of the circularizable padlock that triggers the amplification. It is recommended to add a spacer between the aptamer and the primer to secure that the target binding and padlock hybridization occur simultaneously. Unfortunately, this makes the synthetic strand quite long, precluding the use of the long aptamer. A fluorescein-tagged short reporting strand, whose sequence corresponds to a region of the padlock complementary to the nascent strand, allows the incorporation of thousands of enzymes per aptamer, and thus the signal amplification.

The binding curve obtained with the short aptamer elongated with the RCA primer is shown in Figure 4b and compared with the non-RCA amplified one. Again, a dramatic increase in the current is observed, which is ascribed to the multilabeling of the elongated aptamer. Fitting to the Langmuir equation yields an apparent K_d_ of 188 ± 33 nM. The improvement in affinity is modest (about 6-fold) supporting that the affinity gain depends on the aptamer-target pair.

## 4. Discussion

The affinity of the aptamer for its ligand is a crucial parameter to evaluate its potential utility in diagnostic applications. However, characterization of the binding ability must be carefully conducted in order not to overestimate its performance. In this sense, the use of several techniques makes more robust and reliable the final value, usually in terms of dissociation constant. In this work, we have examined two DNA aptamers derived against the tumor biomarker AFP. Their affinity differs in more than two orders of magnitude. In our hands and using electrochemical detection after aptamer binding on AFP-modified MBs, both aptamers show modest affinity, in the sub-micromolar range. The K_d_ for the short aptamer agrees well with the reported by CE, that is, in solution. However, Dong et al. estimated a much lower value, around 19 nM, when using a sandwich assay. In that experiment, the aptamer was used as a capture element and an antibody as a reporter one. In an analogue experiment, the long aptamer was tested obtaining a K_d_ of 17 nM, almost one order of magnitude higher than the original value, 2.37 nM [10]. The observation of similar K_d_ with two different aptamers and an identical antibody might point to the non-negligible influence of the latter in the value measured, which could result in an overestimation of the K_d_ for the weaker aptamer. If the K_d_ value estimated by the sandwich assay for the short aptamer was true, this would mean that the heterogeneous K_d_ would be one and a half orders of magnitude smaller than the homogeneous one. This is not usually true because of the lack of steric hindrance in solution. Nonetheless, when surfaces with a high load of receptor are used, avidity might occur. For this reason, to estimate the true affinity high loading is not recommended [32].

When estimating the K_d_ by using the Hill model, it is important to verify that the assumptions implicit in the model are met. Of special relevance is to be sure that the concentration of the partner in solution is not affected by the binding; that is, it can be considered constant. If this is not the case, the true concentration of the partner in solution should be precisely calculated. From our experiments, depletion of the aptamer in solution is not observed, so the Hill model can be applied.

The analytical sensitivity can be improved by coupling isothermal DNA amplification strategies. We have demonstrated that both TdT and RCA can enhance the analytical signal. For example, the current improvement for the long aptamer at 500 nM was 7.8-fold when TdT was used. The RCA showed much higher amplification, about 24-fold for the same aptamer concentration. Interestingly, the improvement in the apparent dissociation constant was higher with TdT amplification, 16-fold versus the 6-fold for RCA. We attribute this fact to the different aptamers used. The shift of the binding curves seems to be affinity dependent. The better the affinity, the larger the shift is. In any case, even with the TdT amplification, it was not possible to obtain K_d_ values as low as those previously reported for the aptamer that shows the highest affinity for AFP.

## 5. Conclusions

In this work, we have studied the affinity of two anti-AFP aptamers previously reported to have very different affinities. Using a heterogeneous direct binding assay with electrochemical detection, different from the techniques used to evaluate the affinity in the original work, similar binding constants were estimated for both receptors. However, they are rather poor for analytical applications. By taking advantage of the nucleic acid character of the aptamers, we demonstrated that DNA amplification of the aptamer enables an improvement in sensitivity and in the apparent dissociation constant. DNA amplification strategies can enhance the performance of weak binding aptamers but their magnitude depends on the true affinity. These results are of general significance, as a number of electrochemical biosensors relying on the recognition by poor aptamers could benefit from these results.

## Supplementary Materials

The following are available online at https://www.mdpi.com/2079-6374/10/5/46/s1. The reagents and instrumentation and Table S1: Sequences of the aptamers and other DNA probes used in this work.
