# Supplementary material for: On the Electrochemical Detection of Alpha-Fetoprotein Using Aptamers: DNA Isothermal Amplification Strategies to Improve the Performance of Weak Aptamers
Source: Biosensors (Basel). 2020 Apr 30;10(5):46. doi: 10.3390/bios10050046 (PMC7277757; doi:10.3390/bios10050046)
Supplement: Supplementary file 1 [file biosensors-10-00046-s001.pdf]

biosensors

MDPI

Supplementary Material

On the Electrochemical Detection of Alpha-Fetoprotein Using Aptamers: DNA Isothermal Amplification Strategies to Improve the Performance of Weak Aptamers

Ramón Lorenzo-Gómez [1,2], Daniel González-Robles [1], Rebeca Miranda-Castro [1,2], Noemí de-los-Santos-Álvarez [1,2] and María Jesús Lobo-Castañón [1,2,*]

[1] Dpto. Química Física y Analítica, Universidad de Oviedo, Av. Julián Clavería 8, 33006 Oviedo, Spain; lorenzoramon@uniovi.es (R.L.-G.); dgonrob88@gmail.com (D.G.-R.); mirandarebeca@uniovi.es (R.M.-C.); santosnoemi@uniovi.es (N.S.-Á.)

[2] Instituto de Investigación Sanitaria del Principado de Asturias, Avenida de Roma, 33011 Oviedo, Spain

* Correspondence: mjlc@uniovi.es; Tel.: +34-98-510-6235

Received: 9 April 2020; Accepted: 28 April 2020; Published: date

1. Reagents

Ethylenediaminetetraacetic acid (EDTA) disodium salt dihydrate, sodium dihydrogen phosphate dihydrate, disodium hydrogen phosphate, sodium chloride, ammonium sulfate, 2′-deoxyadenosine-5′-triphosphate (dATP) sodium salt solution, 10× phosphate buffered saline solution (10× PBS), 20× sodium-saline phosphate-EDTA solution (20× SSPE), Tween 20 (70%), bovine serum albumin (BSA), biotin, and 3,3′,5,5′-tetramethylbenzidine (TMB) in a ready-to-use reagent format were purchased from Sigma-Aldrich (Spain). Casein buffer (1%) in 1× PBS, streptavidin-peroxidase conjugate (SA-POD), DNA ligase T4 (5 Weiss U µL-1, supplied in 20 Mm Tris-HCl (pH 7.5), 50 mM KCl, 1 mM DTT, 0.1 mM EDTA and 50% (v/v) glycerol) with its 10× T4 DNA ligase buffer (400 mM Tris-HCl, 100 mM MgCl2, 100 mM DTT, 5 mM ATP), and phi29 DNA polymerase (10 U μL^{-1}, supplied in 50 mM Tris-HCl (pH 7.5), 0.1 mM EDTA, 1 mM DTT, 100 mM KCl, 0.5% (v/v) Nonidet P40, 0.5% (v/v) Tween 20 and 50% (v/v) glycerol) with its 10× reaction buffer (330 mM Tris-acetate (pH 7.9 at 37 °C), 100 mM Mg-acetate, 660 mM K-acetate, 1% (v/v) Tween 20, 10 mM DTT), Dynabeads? M-280 Tosylactivated and Dynabeads? MyOne™ Streptavidin C1 were obtained from Thermo Fisher Scientific (Spain).

Human alpha-fetoprotein (AFP) purified from pooled human cord serum (pro-406) was supplied by ProSpec (Israel), N6-(6-amino)hexyl-2′-deoxyadenosine-5′-triphosphate-biotin triethylammonioum salt (biotin-dATP) by Jena Bioscience GmbH (Germany), deoxyribonucleosides triphosphate (dNTPs) by Bioline (Spain), anti-fluorescein-Fab-fragmentperoxidase conjugate (antiF-POD) by Roche diagnostics (Spain), 5× Bradford reagent by SERVA Electrophoresis GmbH (Germany), SimplySafe DNA staining dye by EURx (Poland), 20 bp DNA Ladder (dye plus) by Takara Bio Inc. (Japan), 6× DNA gel loading buffer by Novagen, Millipore (Spain), and 5× Tris-Borate-EDTA buffer for molecular biology (5× TBE) was supplied by PanReac – AppliChem (Spain).

Terminal deoxynucleotidyl transferase amplification kit was acquired to New England Biolabs (Massachusetts, USA), including the enzyme (20 U µL-1, supplied in 50 mM K-phosphate (pH 7.3), 100 mM NaCl, 1.43 mM 2-mercaptoethanol, 0.1% (v/v) TritonR X-100, and 50% (v/v) glycerol) with its 10× reaction buffer (500 mM Na-acetate, 200 mM Tris-acetate, 100 mM Mgacetate) and 2.5 mM $CoCl_2$.

Oligonucleotide DNA sequences were obtained purified by HPLC from Metabion (Germany), and their sequences are shown in Table S1. All aptamers and DNA probes stock solutions were

aliquoted and diluted with ultrapure water to appropriate concentrations and stored at −20 °C. Water was purified with a Milli-Q system (Millipore, Spain). All other reagents were of analytical grade.

2. Apparatus

Screen-printed electrochemical cells with 4-mm working electrodes made of C (SPCE, DRP-110) were acquired from Metrohm-DropSens (Oviedo, Spain). Before performing any measurement, the electrodes were rinsed with ethanol and deionized water and dried under a nitrogen stream. Electrochemical measurements were performed with a specific connector (DRPDSC, Metrohm-DropSens) acting as interface between the SPCE and a computer-controlled μ-Autolab type II potentiostat equipped with NOVA 2.1. software (Methrom Autolab). All the potentials are referred to the screen-printed Ag pseudoreference electrode (Ag-SPC).

A DynaMagTM-2 magnet (Thermo Fisher Scientific) was used for all magnetic separations and washings. Incubations that required controlled temperature were carried out in a ThermoMixer? Comfort (Eppendorf, Spain), while room temperature incubations were performed in a Dynabeads™ MX 12-tube Mixing Wheel (Thermo Fisher Scientific).

Table S1. Aptamers and oligonucleotides used in this work.

Aptamer /Probe	Sequence (5′→ 3′)
AFP-L-FITC	FITC-GGC AGG AAG ACA AAC AAG CTT GGC GGC GGG AAG GTG TTT AAA TTC CCG GGT CTG CGT GGT CTG TGG TGC TGT
AFP-L-TdT	GGC AGG AAG ACA AAC AAG CTT GGC GGC GGG AAG GTG TTT AAA TTC CCG GGT CTG CGT GGT CTG TGG TGC TGT TTT TTT
AFP-S-FITC	FITC-TCA GGT GCA GTT CTC GAC TCG GTC TTG ATG TGG GT
AFP-S-Biotin	Biotin- TCA GGT GCA GTT CTC GAC TCG GTC TTG ATG TGG GT
AFP-S-TdT	TTT TTT TTT TTC AGG TGC AGT TCT CGA CTC GGT CTT GAT GTG GG
AFP-S-RCA	TCA GGT GCA GTT CTC GAC TCG GTC TTG ATG TGG GT*T TTT TTT TTT* **AGA TAG TAA GTG CAA TCT**
Padlock	**phosphate-TTA CTA TCT** T*AA CAA CAT GAA GAT TGT A*GG TCA GAA CTC ACC TGT TAG AAA CTG TGA AGA TCG CTT **AGA TTG CAC**
Reporter probe	6-FAM-AAC AAC ATG AAG ATT GTA
T40	Biotin-TTT TTT TTT TTT TTT TTT TTT TTT TTT TTT TTT TTT TTT T

Bold nucleotides indicate the region of padlock that hybridized with the extension (primer) of the aptamer. Nucleotides underlined in italics indicate the region that, once copied, hybridizes with the reporter probe.

© 2020 by the authors. Licensee MDPI, Basel, Switzerland. This article is an open access article distributed under the terms and conditions of the Creative Commons Attribution (CC BY) license (http://creativecommons.org/licenses/by/4.0/).
